# Visualization of White Matter Tracts Using a Non-Diffusion Weighted Magnetic Resonance Imaging Method: Does Intravenous Gadolinium Injection Four Hours Prior to the Examination Affect the Visualization of White Matter Tracts?

**DOI:** 10.1371/journal.pone.0091860

**Published:** 2014-03-12

**Authors:** Masahiro Yamazaki, Shinji Naganawa, Hisashi Kawai, Mitsuru Ikeda, Kiminori Bokura, Haruo Isoda, Tsutomu Nakashima

**Affiliations:** 1 Department of Radiology, Nagoya University Graduate School of Medicine, Nagoya, Japan; 2 Department of Radiological Technology, Nagoya University Graduate School of Medicine, Nagoya, Japan; 3 Department of Otorhinolaryngology, Nagoya University Graduate School of Medicine, Nagoya, Japan; University of California at San Francisco, United States of America

## Abstract

**Objectives:**

Visualization of white matter (WM)-tracts such as the corticospinal tract (CST), medial lemniscus (ML), and superior cerebellar peduncle (SCP) using delayed enhanced (DE)-heavily T2-weighted three-dimensional fluid-attenuated inversion-recovery (hT2w-3D-FLAIR) imaging has recently been reported. In that report, all patients were clinically suspected of having Ménière’s disease, because DE-hT2w-3D-FLAIR imaging of the inner ear has been reported to separately visualize perilymph and endolymph fluid and can identify the presence of endolymphatic hydrops. Therefore, the previous report could not rule out the possible effect of delayed enhancement. From this perspective, the purpose of this study was to elucidate if the use of gadolinium affects the visualization of WM-tracts on hT2w-3D-FLAIR.

**Materials and Methods:**

The records of nine patients with suspected Ménière’s disease who underwent plain (P) and DE-hT2w-3D-FLAIR by 3-Tesla were retrospectively analyzed. The regions of interest were set on the CST, ML, and SCP, and on contiguous brain parenchyma: The thalamus (Th), pontine parenchyma (PP), and cerebellar parenchyma (CP), respectively. The signal intensity ratio between each WM-tract and the relevant contiguous brain parenchyma was calculated for both P- and DE-hT2w-3D-FLAIR images, and statistically compared using paired *t*-tests.

**Results:**

The CST/Th signal intensity ratio was 3.75±0.67 on P-hT2w-3D-FLAIR and 3.62±0.50 on DE-hT2w-3D-FLAIR (p = 0.24). The ML/PP signal intensity ratio was 2.19±0.59 on P-hT2w-3D-FLAIR and 2.08±0.53 on DE-hT2w-3D-FLAIR (p = 0.25). The SCP/CP signal intensity ratio was 4.08±0.91 on P-hT2w-3D-FLAIR and 4.04±0.96 on DE-hT2w-3D-FLAIR (p = 0.43). There were no significant differences in the signal intensity ratios between P- and DE-hT2w-3D-FLAIR images.

**Conclusions:**

The use of gadolinium is not necessary for visualization of WM-tracts using hT2w-3D-FLAIR, and P-hT2w-3D-FLAIR without gadolinium may have future clinical applications as an imaging procedure.

## Introduction

Three-dimensional fluid-attenuated inversion recovery (3D-FLAIR) imaging can minimize the undesired ghosts of fluid flow [1] and enable recognition of the subtle compositional changes and the contrast effect in the lymph fluid of the inner ear [2]–[4]. In addition, 3D-FLAIR imaging performed 24 h after an intratympanic gadolinium injection [5]–[10] or 4 h after a double-dose intravenous gadolinium injection [9]–[12] can separately visualize perilymph and endolymph fluid. These imaging techniques using gadolinium can identify the presence of endolymphatic hydrops, or enable evaluation of the characteristics of inner ear pharmacokinetics. Furthermore, heavily T2-weighted 3D-FLAIR (hT2w-3D-FLAIR) imaging is more sensitive than conventional 3D-FLAIR imaging for detecting low concentrations of gadolinium in fluid, and enables visualization of endolymphatic hydrops in patients with Ménière’s disease when performed 4 h after a single-dose intravenous gadolinium injection [13], [14].

**Figure 1:**
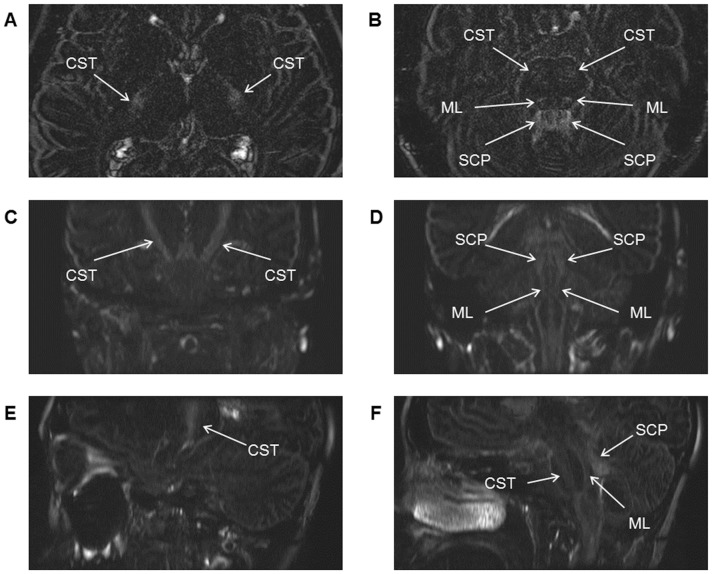
White matter tracts visualized on delayed enhanced-heavily T2-weighted three-dimensional fluid-attenuated inversion recovery (DE-hT2w-3D-FLAIR) images. Axial (**A** and **B**: the thalamic and the pontine levels), coronal (**C** and **D**: the basilar part and the tegmentum of the pons levels), and sagittal (**E** and **F**: the left cerebellar hemisphere and the left side of the pons levels) delayed enhanced-heavily T2-weighted three-dimensional fluid-attenuated inversion recovery (DE-hT2w-3D-FLAIR) images of a single patient who was clinically suspected of having Ménière’s disease are presented. Images were obtained 4 h after a single-dose intravenous gadolinium injection (0.2 mL (0.1 mmol)/kg body weight). Coronal and sagittal images were reconstructed from axial based three-dimensional images (voxel size, 0.5×0.5×1.0 mm). The corticospinal tract (CST), medial lemniscus (ML), and superior cerebellar peduncle (SCP) are visualized clearly and continuously as high-intensity areas. The general parameters for these images were as follows: repetition time, 9000 ms; echo time, 544 ms; inversion time, 2250 ms; number of excitations (NEX), 4.

Although the primary purpose of delayed enhanced (DE)-hT2w-3D-FLAIR imaging obtained 4 h after an intravenous gadolinium injection was to separately visualize perilymph and endolymph fluid and to identify the presence of endolymphatic hydrops using a single-dose (not a double-dose) intravenous gadolinium injection, a recent study revealed that particular white matter (WM)-tracts of the brain such as the corticospinal tract (CST), medial lemniscus (ML), and superior cerebellar peduncle (SCP) were visualized clearly and continuously as areas of high signal intensity on high resolution DE-hT2w-3D-FLAIR images, secondarily (**Figure 1**) [15]. This indicates that hT2w-3D-FLAIR can provide useful supplementary information to diffusion weighted imaging (DWI) for the investigation of WM-tracts. However, this recent study [15] could not completely exclude the possibility that delayed enhancement influenced the visualization of WM-tracts on the hT2w-3D-FLAIR images, because the images were obtained 4 h after intravenous gadolinium injection. The visualization of WM-tracts on hT2w-3D-FLAIR images obtained without gadolinium (plain (P)-hT2w-3D-FLAIR images), would be beneficial for diagnosis, would avoid potential side effects of contrast media, and would be cheaper to administer than images requiring contrast media. However, no report has statistically compared the visualization of WM-tracts between P- and DE-hT2w-3D-FLAIR images. Therefore, the purpose of the present study was to statistically compare the visualization of WM-tracts between P- and DE-hT2w-3D-FLAIR images.

## Materials and Methods

### Ethics Statement

Written informed consent was obtained from all patients and the Ethics Review Committee of Nagoya University Graduate School of Medicine approved the study.

### Study Population

The records of nine consecutive adult patients (five male, four female; aged 27–74 years; mean age, 49.1 years) who underwent P- and DE-hT2w-3D-FLAIR imaging of the head at our institute between December 2010 and February 2011 were retrospectively analyzed. In all patients, the two exams were conducted on the same day. All patients were clinically suspected of having Ménière’s disease, and they underwent magnetic resonance imaging to determine whether signal intensity changes of inner ear lymph fluid or endolymphatic hydrops were present. Initially, all patients underwent hT2w-3D-FLAIR imaging before gadolinium injection (P-imaging). Subsequently, all patients received intravenous administration of a single-dose (0.2 mL (0.1 mmol)/kg body weight) of gadolinium-diethylenetriamine pentaacetic acid-bis (methylamide) (Omniscan, Daiichi Sankyo Co., Ltd., Tokyo, Japan) and underwent hT2w-3D-FLAIR imaging 4 h after injection (DE-imaging). In healthy subjects, a delay of 4 h between intravenous gadolinium injection and 3D-FLAIR imaging is optimal for wide distribution of gadolinium in the lymphatic space of the labyrinth [16].

### Imaging Protocol

All scans were performed with a 3-Tesla magnetic resonance imaging system (Magnetom Verio; Siemens AG, Erlangen, Germany) using a receive-only, 32-channel, phased-array coil. The parameters for hT2w-3D-FLAIR imaging were as follows: sampling perfection with application-optimized contrast using different flip angle evolution (SPACE)-based sequences with a frequency-selective fat-suppression pre-pulse; repetition time, 9000 ms; echo time, 546 ms; inversion time, 2350 ms; initial refocusing flip angle, 180°, rapidly decreased to constant flip angle, 120° for the turbo spin-echo refocusing echo train in the SPACE sequences; echo train length, 105; matrix size, 267×320; 112 axial, 0.8-mm-thick slices covering from the body of the lateral ventricle to the upper cervical spinal cord, including the labyrinth, with a 172.5×206 mm field of view; generalized autocalibrating partially parallel acquisition (GRAPPA) [17] acceleration factor, 3; voxel size, 0.6×0.6×0.8 mm; number of excitations (NEX), 2; scan time, 5 min 26 s; readout bandwidth, 601 Hz/pixel; echo spacing, 4.34 ms.

### Imaging Evaluation

**Figure 2:**
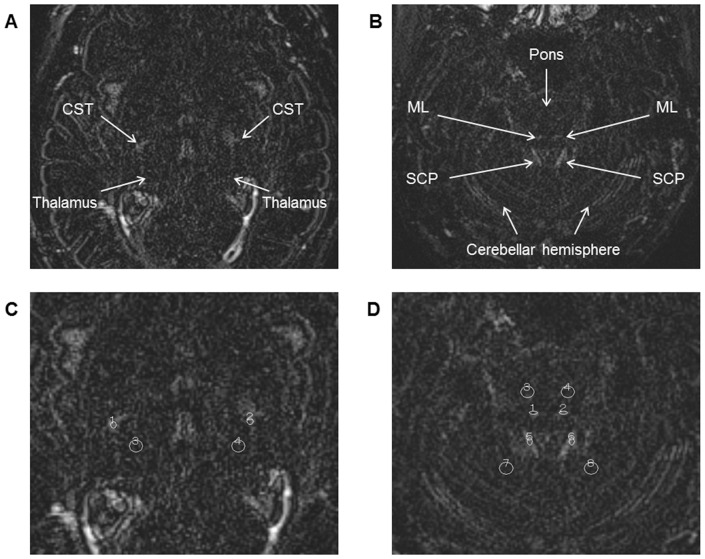
The regions of interest setting on plain-heavily T2-weighted three-dimensional fluid-attenuated inversion recovery (P-hT2w-3D-FLAIR) images. Axial, plain, heavily T2-weighted three-dimensional fluid-attenuated inversion recovery (hT2w-3D-FLAIR) images obtained from a single patient without gadolinium injection. Images on the left side (**A** and **C**) are at the level of the thalamus (Th) and images on the right side (**B** and **D**) are at the level of the trigeminal nerve. The corticospinal tract (CST), medial lemniscus (ML), and superior cerebellar peduncle (SCP) are visualized as areas of high signal intensity (**A** and **B**). Images **C** and **D** are trimmed magnifications of images **A** and **B**, with regions of interest (ROI) setting. Oval 5-mm^2^ ROI were set on the CST, ML, SCP, and circular 15-mm^2^ ROI were set on the Th, pontine parenchyma (PP), and cerebellar parenchyma (CP) (**C** and **D**). To evaluate the signal contrast between white matter (WM)-tracts and contiguous brain parenchyma, the following signal intensity ratios between WM-tracts and contiguous brain parenchyma were calculated: the ratio of the signal intensity of the CST to the signal intensity of the Th on the same side (CThR), the ratio of the signal intensity of the ML to the signal intensity of the PP on the same side (MPPR), and the ratio of the signal intensity of the SCP to the signal intensity of the CP on the same side (SCPR).

**Figure 3:**
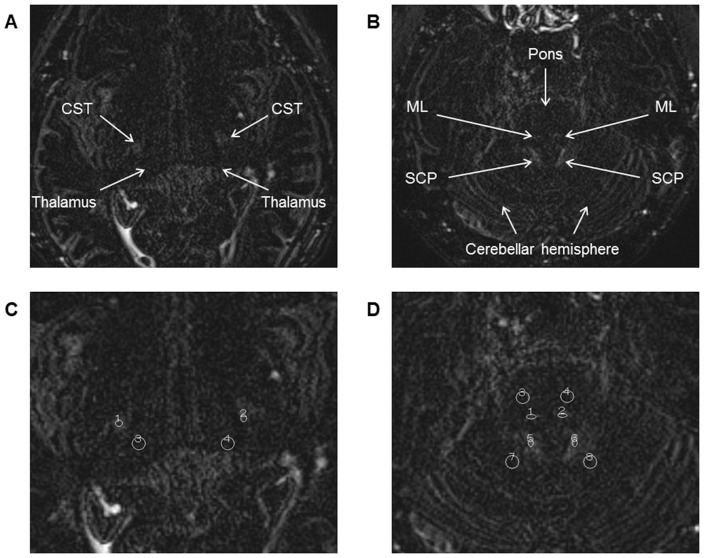
The regions of interest setting on delayed enhanced-heavily T2-weighted three-dimensional fluid-attenuated inversion recovery (DE-hT2w-3D-FLAIR) images. Axial, delayed enhanced, heavily T2-weighted three-dimensional fluid-attenuated inversion recovery (hT2w-3D-FLAIR) images from the same patient as in **Figure 2**. Images were obtained 4 h after intravenous gadolinium injection. Images on the left side (**A** and **C**) are at the levels of the thalamus (Th), and images on the right side (**B** and **D**) are at the level of the trigeminal nerve. The corticospinal tract (CST), medial lemniscus (ML), and superior cerebellar peduncle (SCP) are visualized as areas of high signal intensity (**A** and **B**). Images **C** and **D** are trimmed magnifications of images **A** and **B**, with regions of interest (ROI) setting. Oval 5-mm^2^ ROI were set on the CST, ML, SCP, and circular 15-mm^2^ ROI were set on the Th, pontine parenchyma (PP), and cerebellar parenchyma (CP) (**C** and **D**). To evaluate the signal contrast between white matter (WM)-tracts and contiguous brain parenchyma, the following signal intensity ratios between WM-tracts and contiguous brain parenchyma were calculated: the ratio of the signal intensity of the CST to the signal intensity of the Th on the same side (CThR), the ratio of the signal intensity of the ML to the signal intensity of the PP on the same side (MPPR), and the ratio of the signal intensity of the SCP to the signal intensity of the CP on the same side (SCPR).

The images were analyzed on a workstation (Rapideye Station; Toshiba Medical Systems Corporation, Otawara, Japan). Oval 5-mm^2^ regions of interest (ROI) were set on the CST, ML, SCP, and circular 15-mm^2^ ROI were set on the relevant contiguous brain parenchyma: The thalamus (Th), pontine parenchyma (PP), and cerebellar parenchyma (CP), respectively (**Figure 2** and **Figure** **3**). The ROI on the CST and Th were set at the level of the Th, and the ROI on the ML, SCP, PP and CP were set at the level of the trigeminal nerve. Each ROI was set on both the right and the left sides of each image.

To evaluate the signal contrast between WM-tracts and contiguous brain parenchyma, signal intensity ratios between the WM-tract and the contiguous brain parenchyma were defined and calculated as follows: CThR, the ratio of the signal intensity of the CST to the signal intensity of the Th on the same side; MPPR, the ratio of the signal intensity of the ML to the signal intensity of the PP on the same side; SCPR, the ratio of the signal intensity of the SCP to the signal intensity of the CP on the same side. These ratios were calculated for both P- and DE-hT2w-3D-FLAIR images. In addition, the location and the size of each WM-tract on axial images were also measured. The location of each WM-tract was calculated as follows: CST, lateral distance from the lateral margin of the third ventricle at the thalamic level; ML, anterior distance from the anterior margin of the fourth ventricle at the trigeminal nerve level; and SCP, lateral distance from the lateral margin of the fourth ventricle at the trigeminal nerve level. The size of each WM-tract was calculated by surrounding the margin of high intensity area: CST, at the thalamic level; ML and SCP, at the trigeminal nerve level. These values were calculated for both P- and DE-hT2w-3D-FLAIR images. The existence of motion artifact was assessed visually, at the same time as the WM-tract analysis.

### Statistical Analysis

Paired *t*-tests were used to compare CThR, MPPR, SCPR, the location and the size of each WM-tract between P- and DE-hT2w-3D-FLAIR images. The level of significance was set at p<0.05.

## Results

**Figure 4:**
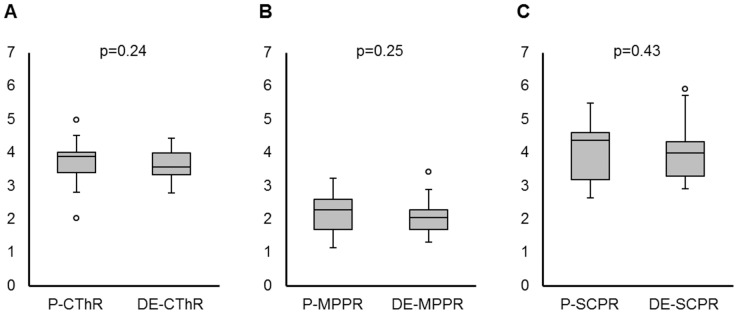
The signal intensity ratios of the white matter tracts to the contiguous brain parenchyma. **A)** The ratio of the signal intensity of the corticospinal tract to the signal intensity of the thalamus on the same side on plain (P-CThR) and delayed enhanced (DE-CThR) heavily T2-weighted three-dimensional fluid-attenuated inversion recovery (hT2w-3D-FLAIR) images. The P-CThR mean was 3.75±0.67 and the DE-CThR mean was 3.62±0.50 (p = 0.24, n = 18). There was no significant difference between P- and DE-CThR. **B)** The ratio of the signal intensity of the medial lemniscus to the signal intensity of the pontine parenchyma on the same side on plain (P-MPPR) and delayed enhanced (DE-MPPR) hT2w-3D-FLAIR images. The P-MPPR mean was 2.19±0.59 and the DE-MPPR mean was 2.08±0.53 (p = 0.25, n = 18). There was no significant difference between P- and DE-MPPR. **C)** The ratio of the signal intensity of the superior cerebellar peduncle to the signal intensity of the cerebellar parenchyma on the same side on plain (P-SCPR) and delayed enhanced (DE-SCPR) hT2w-3D-FLAIR images. The P-SCPR mean was 4.08±0.91 and the DE-SCPR mean was 4.04±0.96 (p = 0.43, n = 18). There was no significant difference between P- and DE-SCPR.

No images from any patients showed motion artifact. The signal intensity ratios for each patient are shown in **Table 1**. The CThR was 3.75±0.67 on P-hT2w-3D-FLAIR images and 3.62±0.50 on DE-hT2w-3D-FLAIR images (p = 0.24, n = 18, **Figure 4** **A**). The MPPR was 2.19±0.59 on P-hT2w-3D-FLAIR images and 2.08±0.53 on DE-hT2w-3D-FLAIR images (p = 0.25, n = 18, **Figure 4** **B**). The SCPR was 4.08±0.91 on P-hT2w-3D-FLAIR images and 4.04±0.96 on DE-hT2w-3D-FLAIR images (p = 0.43, n = 18, **Figure 4** **C**). There were no significant differences between P- and DE-hT2w-3D-FLAIR images for any ratio. In addition, the location and the size of each WM-tract on axial hT2w-3D-FLAIR images are shown in **Table 2**. There were no significant differences between P- and DE-hT2w-3D-FLAIR images for the location and the size of each WM-tract.

**Table 1:** The signal intensity ratios on hT2w-3D-FLAIR images for each patient.

Pt no.	Side	Age	Sex	P-CThR	DE-CThR	P-MPPR	DE-MPPR	P-SCPR	DE-SCPR
1	L	27	F	4.99	3.92	2.22	1.30	4.19	4.27
1	R	27	F	3.82	2.96	1.36	1.75	5.00	5.51
2	L	74	F	3.67	4.01	1.61	1.53	4.05	4.34
2	R	74	F	3.96	3.32	1.49	1.67	2.97	4.09
3	L	34	M	4.28	3.56	2.85	2.24	4.60	4.04
3	R	34	M	3.89	4.29	2.61	2.90	4.54	3.77
4	L	66	F	3.18	3.46	2.10	3.44	3.98	3.62
4	R	66	F	3.35	4.23	3.01	2.08	5.48	4.95
5	L	31	F	2.05	2.77	2.34	1.69	4.94	5.70
5	R	31	F	4.41	3.81	2.35	1.61	4.55	5.92
6	L	67	M	2.80	3.20	3.21	2.29	5.26	3.16
6	R	67	M	3.24	4.42	2.50	1.63	4.60	2.90
7	L	60	M	3.97	3.40	2.09	2.20	3.16	4.09
7	R	60	M	4.01	3.58	2.73	2.53	4.58	2.96
8	L	50	M	3.93	4.32	1.14	2.00	2.88	2.94
8	R	50	M	3.87	3.39	1.67	1.94	3.26	2.91
9	L	33	M	3.52	3.61	1.77	2.12	2.84	3.66
9	R	33	M	4.51	2.91	2.35	2.53	2.63	3.92
**Average ± SD**				**3.75±0.67**	**3.62±0.50**	**2.19±0.59**	**2.08±0.53**	**4.08±0.91**	**4.04±0.96**

*hT2w-3D-FLAIR:* heavily T2-weighted three-dimensional fluid-attenuated inversion-recovery, *Pt no.:* patient number, *P:* plain (i.e., without gadolinium injection), *DE:* delayed enhancement (4 hours after intravenous gadolinium injection), *CThR:* ratio of the signal intensity of the corticospinal tract to the signal intensity of the thalamus on the same side, *MPPR:* ratio of the signal intensity of the medial lemniscus to the signal intensity of the pontine parenchyma on the same side, *SCPR:* ratio of the signal intensity of the superior cerebellar peduncle to the signal intensity of the cerebellar parenchyma on the same side, *L:* left, *R:* right, *F:* female, *M:* male, *SD:* standard deviation.

**Table 2:** The location and the size of each WM-tract on axial hT2w-3D-FLAIR images.

	P (Average ± SD)	DE (Average ± SD)	p-value
**CST-distance (mm)** a	20.03±0.58	20.00±0.52	p = 0.42
**CST-size (mm^2^)** b	53.62±3.66	53.35±4.41	p = 0.33
**ML-distance (mm)** a	6.20±0.33	6.23±0.31	p = 0.59
**ML-size (mm^2^)** b	10.11±1.04	9.95±1.00	p = 0.28
**SCP-distance (mm)** a	0^c^	0^c^	–
**SCP-size (mm^2^)** b	48.37±5.98	48.44±5.47	p = 0.53

*WM-tract:* white matter tract, *hT2w-3D-FLAIR:* heavily T2-weighted three-dimensional fluid-attenuated inversion-recovery, *P:* plain (i.e., without gadolinium injection), *DE:* delayed enhancement (4 hours after intravenous gadolinium injection), *SD:* standard deviation, *CST:* corticospinal tract, *ML:* medial lemniscus, *SCP:* superior cerebellar peduncle.

a The location of each WM-tract was calculated as follows: CST, lateral distance from the lateral margin of the third ventricle at the thalamic level; ML, anterior distance from the anterior margin of the fourth ventricle at the trigeminal nerve level; and SCP, lateral distance from the lateral margin of the fourth ventricle at the trigeminal nerve level.

b The size of each WM-tract was calculated by surrounding the margin of high intensity area: CST, at the thalamic level; ML and SCP, at the trigeminal nerve level.

c SCP was just lateral to the fourth ventricle at the trigeminal nerve level in all patients.

## Discussion

The present study is the first statistical comparison of the visualization of certain WM-tracts between P- and DE-hT2w-3D-FLAIR images. The results revealed that delayed enhancement of a gadolinium contrast agent did not improve the visualization of the CST, ML and SCP on hT2w-3D-FLAIR images. The intact blood-brain barrier prevents the passage of larger molecules from the systemic circulation to the brain parenchyma [18]–[20], and the results of the present study indicate that the intravenously injected gadolinium contrast agent had not passed through the intact blood-brain barrier 4 h after injection.

Previous studies have reported that WM-tracts such as the CST are visualized as high-intensity regions on two-dimensional (2D)-spin-echo (SE)-FLAIR and 2D-SE-T2-weighted images [21], [22]. In these studies, the high signal intensity of particular WM-tracts was thought to arise from structural features of the WM-tracts such as unmyelinated or sparsely myelinated fibers within the tracts [21], or the presence of large fibers with thick myelin sheaths [22]. It is unlikely that the visualization of WM-tracts on 3D-FLAIR images obtained using varying flip angles is related to noticeable inherent diffusion sensitivity [23]. In a recent study, the CST, ML, and SCP were visualized clearly and continuously on hT2w-3D-FLAIR images, although other WM-tracts, such as the middle cerebellar peduncle, were not clearly visualized [15]. Taken together, this indicates that the high signal intensity of certain WM-tracts on hT2w-3D-FLAIR images is likely due to T2 differences caused by the structural features of particular WM-tracts, rather than to the effect of diffusion related to the direction of the nerve fibers.

At the present moment, methodologies for visualizing or analyzing WM-tracts are generally based on DWI techniques, including diffusion tensor imaging and fiber tracking [24]–[28]. However, these DWI techniques have some weak points, including low spatial resolution, frequent image distortion arising from magnetic susceptibility, kissing fibers, crossing fibers, branching fibers, and the effects of arbitrariness of the threshold settings [29], [30]. The visualization and analysis of WM-tracts using hT2w-3D-FLAIR images have some advantages over DWI, including the availability of 3D-images, high resolution, non-echo-planar image sequences, and less image distortion due to magnetic susceptibility. In addition, from the results of the present study, the signal intensity, the location and the size of particular WM-tracts could be measured on P-hT2w-3D-FLAIR. Therefore, hT2w-3D-FLAIR imaging can provide useful supplementary information to existing DWI based techniques. This may be useful for investigation of WM-tracts in degenerative disorders, or preoperative estimation of WM-tract pathways.

Previous reports have revealed that proton density weighted imaging (PDWI) also provides good differentiation between gray and white matter throughout the brain and including the brain stem [31]–[33]. On PDWI, WM-tracts were visualized as low signal intensity areas. On the other hand, compared with PDWI, the WM-tracts on hT2w-3D-FLAIR were visualized as high signal intensity areas. This positive signal means that WM-tracts visualized on hT2w-3D-FLAIR should be less influenced by susceptibility artifacts, although a 3-Tesla scanner was used in the present study. In addition, the visualization and analysis of WM-tracts using hT2w-3D-FLAIR images have an advantage of the availability of high resolution 3D-images, compared with above mentioned reports about PDWI.

It has recently been reported that visualization of WM-tracts of the brain stem is better on conventional 3D-FLAIR images than on 2D-FLAIR and 2D-T2-weighted images [34]. The spatial resolution of images in the present study was higher than in this previous report. In addition, the results of the present study newly revealed that the visualization and the measurements of the location and the size of particular WM-tracts on hT2w-3D-FLAIR images does not require delayed enhancement of a gadolinium contrast agent, and indicates that the clinical application of hT2w-3D-FLAIR imaging without gadolinium injection is possible. Furthermore, the visibility of WM-tracts, or the contrast between WM-tracts and the surrounding structures, on hT2w-3D-FLAIR images may surpass that observed on conventional 3D-FLAIR images. This is an important comparative research issue that must be elucidated in future investigations.

The injection of intravenous gadolinium contrast agents carries a risk of nephrogenic systemic fibrosis [35]–[37], and drug injections also have the potential to cause allergic reactions, or some unknown side effects. The results of the present study revealed that gadolinium contrast agents are not required for the visualization of certain WM-tracts on hT2w-3D-FLAIR images. This is meaningful, and may have clinical benefits by lowering the risk of the drug side effects.

The present study has some limitations. The study population consisted of a small number of patients with clinically suspected Ménière’s disease, and did not include healthy volunteers. In addition, the imaging protocol applied 2 NEX for obtaining hT2w-3D-FLAIR images, in contrast to the 4 NEX used in a previous report [15]. A low NEX can reduce the signal-to-noise ratio, and the relatively low signal intensity of the ML, which is smaller than the CST and SCP, might be due to this small NEX. Therefore, a higher NEX may be more appropriate for clinical application.

## Conclusion

The present study revealed that visualization of the CST, ML and SCP on hT2w-3D-FLAIR images does not require delayed enhancement of a gadolinium contrast agent, and indicates that the clinical application of hT2w-3D-FLAIR imaging without gadolinium injection is possible. hT2w-3D-FLAIR imaging without gadolinium injection can provide useful supplementary information to existing DWI based techniques for the investigation of WM-tracts.
